# Fog-Edge Collaborative Task Offloading Strategy Based on Chaotic Teaching and Learning Particle Swarm Optimization

**DOI:** 10.1155/2022/3343051

**Published:** 2022-06-28

**Authors:** Songyue Han, Wei Huang, DaWei Ma, JiLian Guo, Hang He

**Affiliations:** ^1^Communications Non-Commissioned Officer School, Army Engineering University of PLA, Chongqing 400035, China; ^2^32705 Unit of PLA, Xi'an, Shaanxi 710086, China; ^3^Air Force Engineering University, Xi'an, Shaanxi 710032, China; ^4^63769 Unit of PLA, Xi'an, Shaanxi 710086, China

## Abstract

To improve the contradiction between the surge of business demand and the limited resources of MEC, firstly, the “cloud, fog, edge, and end” collaborative architecture is constructed with the scenario of smart campus, and the optimization model of joint computation offloading and resource allocation is proposed with the objective of minimizing the weighted sum of delay and energy consumption. Second, to improve the convergence of the algorithm and the ability to jump out of the bureau of excellence, chaos theory and adaptive mechanism are introduced, and the update method of teaching and learning optimization (TLBO) algorithm is integrated, and the chaos teaching particle swarm optimization (CTLPSO) algorithm is proposed, and its advantages are verified by comparing with existing improved algorithms. Finally, the offloading success rate advantage is significant when the number of tasks in the model exceeds 50, the system optimization effect is significant when the number of tasks exceeds 60, the model iterates about 100 times to converge to the optimal solution, the proposed architecture can effectively alleviate the problem of limited MEC resources, the proposed algorithm has obvious advantages in convergence, stability, and complexity, and the optimization strategy can improve the offloading success rate and reduce the total system overhead.

## 1. Introduction

The development of 5G and mobile edge computing (MEC) has driven the convergence and evolution of mobile Internet and IoT services and become the enabling technology for intelligent transformation of many industries in society. As a typical example in the field of education, the smart campus has undergone digitalization, informatization, and intelligent transformation and is in urgent need of upgrading in teaching methods and infrastructure. New teaching applications such as remote mixed reality teaching, ultra-clear live classroom, and video image recognition require large bandwidth, low latency, wide connectivity, and high mobility network bearing, and the traditional central cloud long-distance multi-hop transmission mode obviously cannot meet the demand.

Currently, the combination of cloud computing and edge computing is the main strategy to solve the above problems, and literature [1] proposes a convergence architecture of MCC and cloudlet, and literature [2] proposes a “cloud-edge collaboration” architecture to achieve cloud-edge balancing of task load. However, with the continuous growth of incoming devices and data traffic, MEC servers with limited resources will face problems such as overload and resource competition, resulting in additional latency and energy consumption, which directly affect the end device life cycle and quality of experience (QoE) of services. To solve this problem, there are two main directions of related research: first, at the level of intrinsic mechanism, through offloading decision and resource allocation as the 2 main entry points, with service delay, terminal energy consumption, or both trade-offs as the optimization objectives, respectively, and single (multiple) user and single (multiple) MEC server as the scenario model. In [3], a heuristic algorithm is proposed to solve the joint optimization problem of delay and energy consumption in the telematic scenario based on the game idea; in [4], an adaptive offloading decision based on the multiarmed slot machine theory is proposed to optimize the task delay for the time-varying network topology, task load, and channel state; in [5], a Lyapunov-based online algorithm is applied to solve the optimization problem of the trade-off between energy consumption and delay; and in [6], the trade-off between energy consumption and delay is applied to solve the optimization problem. In [6], a multi-platform intelligent offloading and resource allocation algorithm is proposed with latency as the optimization goal; secondly, at the external architecture level, literature [7] proposes an underwater “cloud-side-end” architecture to solve the data processing of hydroacoustic sensing network, and literature [8] integrates fog computing with 5G network and establishes a “cloud-fog-end” architecture for industrial scenarios. In [8], a “cloud-fog-end” architecture for industrial scenarios is established by integrating fog computing with 5G networks.

However, many computing tasks at the reality level are not suitable for partial offload, and the differences in their data partition types and business attributes themselves can cause time differences in result feedback due to uncertainties in channel quality, network jitter, and task queues, thus affecting task experience and completeness. In addition, while the edge level is oriented to general-scale latency-sensitive applications, the central cloud is oriented to large-volume non-latency-sensitive tasks, while a smooth transition intermediate layer is lacking for tasks that are large in volume and have high latency requirements. Therefore, this study uses regional-level macro-base stations as fog computing nodes to design a system architecture with smooth transition of arithmetic power and latency capability to cope with differentiated mobile Internet services.

Hence, indoor and outdoor linked teaching in the smart campus requires a large number of mobile terminals, IoT devices, and audiovisual collection devices to access the network efficiently and flexibly, and business data are restricted to flow and processing in local, edge, fog nodes, and central cloud according to the security level and computing power requirements, which should also focus on the security of research-sensitive data isolated from the public network while taking into account the issues of mobility, limited resources, and deployment costs. The existing studies mentioned above have also focused on the security of isolating sensitive data from the public network. The existing research mentioned above has less joint optimization on offloading decision and resource allocation at the intrinsic mechanism level and lacks an architecture for smooth descent of arithmetic power gradient at the extrinsic architecture level. To this end, the following innovative work is carried out in this study.

1. Using 5G MEC as the platform base technology, we propose a four-layer teaching evaluation system based on “cloud, fog, edge, and end” architecture for indoor and outdoor linked teaching scenarios and realize the collaborative sharing of cloud, fog, edge, and end.
2. The joint optimization model of offloading decision, communication, and computing resource allocation is proposed with the weighted sum of delay and energy consumption as the optimization objective, and simulation experiments are conducted based on the architecture model to verify the advantages of the proposed architecture and algorithm strategy from the external architecture to the internal mechanism at two levels.
3. The proposed CTLPSO is compared with the existing improved algorithm in terms of convergence speed, accuracy, and complexity using sine chaos mapping for population initialization and segmented inertia weights to enhance the global exploration capability of the algorithm and give it adaptive characteristics.

## 2. Related Work

### 2.1. Cloud, Fog, and Edge Computing

**Figure 1:**
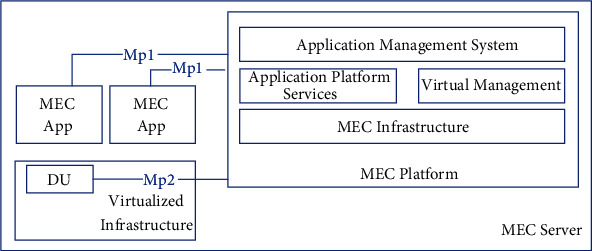
MEC system architecture diagram.

Edge computing broadly refers to the sinking of computing power, storage, and communication resources from the central network to the edge of the network to provide the corresponding services [9], thus achieving the goals of bandwidth saving, latency reduction, load equalization, high isolation, and strong awareness. The MEC system architecture is shown in Figure 1. Its main sponsor, the European Telecommunications Union organization (ETSI), has defined different categories, namely mobile edge computing (MEC) and multi-access edge computing (MEC) for mobile and heterogeneous networks, respectively, and as research progresses, heterogeneous access networks are expected to be extended to non-3GPP networks and other wired networks [10]. Meanwhile, scholars at home and abroad have given the definition of MEC mainly from three levels: data flow [11], network location [12], and cloud computing evolution [13], with different descriptive perspectives but consistent connotations. Computational offloading is one of the core aspects and key technologies for MEC to realize service triage.

**Figure 2:**
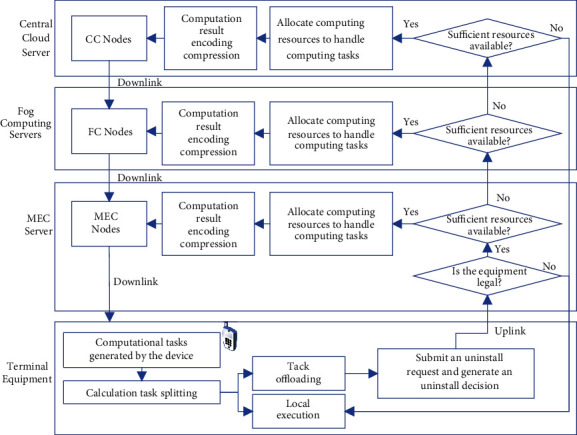
Cloud-fog-edge-terminal collaborative computing offload model.

The broad category of edge computing is specifically realized by three paradigms: fog computing (FC), cloudlet computing (CC), and mobile edge computing (MEC) [14]. The essential logic of the three is the same, but the difference lies in the system architecture, hardware facilities and communication standard, etc. CC is mainly oriented to traditional telecommunication-specific devices; MEC is mainly oriented to mobile communication infrastructure and network architecture. Most of them are general-purpose servers, which are free from traditional telecom equipment constraints, so they can be deployed in physical and virtual forms based on general-purpose servers and do not affect the network architecture; FC has a large radiation range, and the overall computing power resources are much larger than those of MEC, focusing on the relay role between the terminal and the central cloud, and its servers are mostly compatible with special telecom equipment, and its network location is farther from the terminal equipment than that of MEC. It is far away from the end devices, so it is suitable for processing large volume of computing tasks with certain demand for latency. The proposed 4-layer architecture offload model is shown in Figure 2.

### 2.2. Smart Campus

The term “Smart Campus” originates from the concept of “Smart Earth” proposed by IBM in November 2008 and evolves under the support of a series of domestic education informatization policies. Different definitions are given by domestic scholars: Jiang et al. [15] define it from the perspective of information technology support conditions and believe that smart campus is a further deepening and upgrading of digital campus and an advanced form of university informatization; Huang et al. [16] define it from the perspective of learning environment and believe that smart campus should have the ability of environment awareness and customized learning; Wang [17] emphasizes the integration of information technology and pedagogy; the “Smart Campus General Framework” (GB/T/T General Framework for Campus” (GB/T36342-2018) defines a smart campus as an organic interface between physical and information spaces and emphasizes the human access to information resource services as they happen [18]. According to the above definitions, a smart campus should have capability features such as network access on demand, heterogeneous data fusion, cross-platform interaction of systems, and open network capabilities to provide high-quality network support for teaching, research, training, life, and campus governance with access on demand.

### 2.3. Swarm Intelligence Algorithm

Existing studies mostly aim at minimizing the energy consumption under the maximum delay constraint or minimizing the delay under the energy consumption constraint, and existing studies have confirmed that the multi-objective optimization problem is an NP-hard problem [19]. Therefore, in this study, we develop a corresponding optimization strategy based on the swarm intelligence optimization algorithm [20] and improve the classical swarm intelligence algorithm to improve the speed, accuracy, and stability of the convergence of the algorithm while improving the classical swarm intelligence algorithm, which is prone to “premature” problems. In the meantime, we seek to balance the “development” and “exploration” capabilities of the algorithm to improve the effectiveness of the target system.

## 3. Model Construction and Problem Transformation

### 3.1. System Model

**Figure 3:**
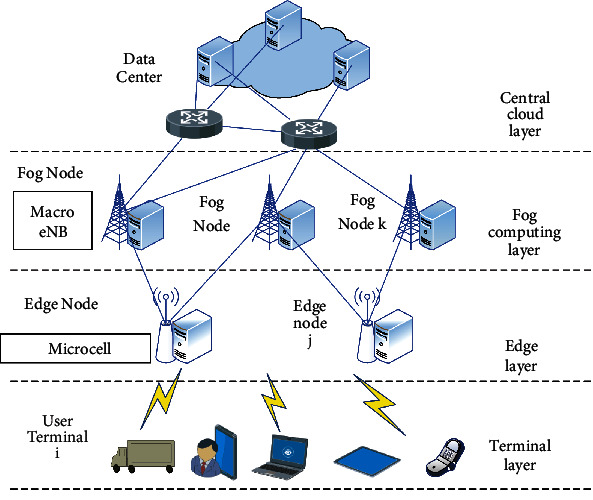
System model.

As the smart campus contains a large number of connected terminals, when the computing resources at the edge layer cannot meet the task computation requirements, the computation tasks offloaded to the edge layer are forwarded to the fog computing layer for processing, and if the resources at the terminal layer, edge layer, and fog computing layer cannot meet the task processing requirements, the tasks are forwarded to the central cloud for processing. Therefore, this study takes the “cloud, fog, edge, and end” architecture in Figure 3 as the information infrastructure of the smart campus and constructs the corresponding computing model, which includes user terminals, various types of base stations, MEC nodes, FC nodes, and cloud computing data centers. Four types of computation nodes are shown in Table 1.

In the model, the relevant equipment and facilities play the following functions.

1. User Terminal: it consists of various types of access terminal devices, including intelligent mobile terminals, IoT devices, and embedded sensing devices, which mainly provide corresponding information services and generate various computing tasks. It also needs to establish communication with the higher-level computing nodes to determine whether to offload and the decision of where to offload.
2. Each Type of Base Station
3. In the target system, the selection of each node base station will be carried out by integrating factors such as service demand, cost budget, deployment cycle, operation and maintenance threshold, and the current status of infrastructure. Overall, the route of smooth evolution of 5G with nonindependent grouping (NSA) to independent grouping (SA) is considered. Among them, the fog computing layer nodes are mainly macro-stations and pole stations, which constitute macro-cells (macro-cell) with coverage radius of 1–25 km, and macro-stations adopt 4.9 or 2.6 GHz band, with bandwidth of 160 M, power of 200–320 W, and channel number of 32T32 R or 64T64 R. The nodes at the edge layer are mainly micro-base stations, which constitute microcell with coverage radius of 30–300 m, and micro-stations are mainly in the 1.8 or 2.6 GHz band with 160 M bandwidth, 2T2R channels, and 2–5 W power; at the terminal layer, portable stations, UAV lift stations, and repeater stations are mainly deployed in combination with carriers such as personnel, vehicles, and UAVs to play the role of mobile blindness filling and hot spot enhancement. In addition, some indoor scenes deploy room substations as appropriate.
4. In particular, micro-stations and portable stations are mainly deployed in the blind spots and hotspots of macro-stations, which are composed of microcell with low transmitting power and support small distance frequency multiplexing and more channels per unit area, thus significantly increasing service density and system capacity, while RF interference is very low; therefore, cells of different capacities are deployed overlappingly, constituting a macro-cell as the upper layer. Therefore, overlapping deployment of cells with different capacities constitutes a multiple cellular system with macro-cell as the upper layer of the network and dense microcell, which makes the whole network system resilience and capacity significantly increased. However, in the three-dimensional multilayer cellular, with the proliferation of frequency-using devices, intra-area or inter-area interference inevitably occurs. At this time, the repeater, as a co-channel amplification device, picks up the signal of the sender antenna in the downlink, isolates the signal outside the bandpass through a bandpass filter, and transmits the filtered signal to the target area again after power amplification, which can effectively alleviate the signal interference problem.
5. MEC Node
6. While the deployment of MEC under 4G architecture mostly requires the introduction of control entities and relatively fixed deployment locations, the deployment of 5G architecture is more flexible, thanks to NFV and SDN technologies on the one hand, and the application of SBA architecture with flexible and tailorable network functions and architecture on the other hand, thanks to the fact that MEC servers are mainly available in two forms: physical servers and virtual packages running on common servers. In addition to allocating computing resources for computing tasks offloaded by terminals, MEC nodes provide location-based customized services based on network context and user information. In addition, it can also provide data triage routing and hot content caching in conjunction with content delivery network (CDN).
7. FC Node
8. There are 3 main categories of fog nodes, which are fog server, fog-edge node, and thin fog in order from high to low according to the resource capacity, and all 3 categories can be deployed in combination with the corresponding information and communication infrastructure, and all have certain computing, storage, and communication capabilities [21]. In the model of Figure 3, the fog nodes are simplified into one class, and the target system in this study chooses a 2-tier fog computing architecture of main fog and thin fog and deploys fog nodes of different complexities on demand. Among them, the fog server is regarded as the main fog node with relatively large computing power and storage resources, but when its resource load is exceeded, the data tasks can be transferred to the central cloud for processing; the fog-edge nodes are regarded as thin fog nodes, which are composed of intelligent gateways, border routers, etc. [22]. Mist nodes are not directly connected to the terminal or the central cloud, but act as middleware for preprocessing tasks and triaging data for intelligent routing.
9. Cloud Computing Data Center
10. The data center not only provides ample computing, storage, and communication resources but also has corresponding business application servers to support institutions to deploy core network equipment for education private networks and realize virtualization dedicated under the same physical resources through network slicing to ensure high network isolation requirements for sensitive data and core-sensitive services in the field of teaching and research.

Based on the foregoing, the modeling is as follows.

In the edge layer, there are user terminal devices, randomly distributed in the edge layer within the cell coverage of the edge nodes, and the computational tasks generated by the user terminals are defined as *T*={*T*_1_, *T*_2_, *T*_3_,…, *T*_*n*_}. There are two attributes of tasks, namely data size and workload, where data size is used for the task offloading process and workload is used for the task computation process. Therefore, the tasks generated by the user terminal are represented by the tuple *T*_*i*_=(*D*_*i*_, *C*_*i*_), *D*_*i*_ is the amount of input data, i.e., the amount of data required to offload the task. *T*_*i*_, *C*_*i*_ is the computing load, that is, the computing resources required to process task*T*_*i*_, expressed in CPU cycles.

Since the model in this study has a 4-layer architecture, when the first 3 layers are unable to complete the task processing, the bulk task is offloaded to the cloud computing center for processing. Usually, such tasks have an extremely high demand for storage and computing power resources and are mostly non-latency-sensitive services, so it is not practical to consider computational latency and energy consumption in the central cloud layer. Therefore, the model is mainly built for the first 3 layers, and to simplify the computational derivation process, offloading decision variables are introduced in the terminal layer and edge layer, respectively, *α* and *β*, *α*, *β* ∈ {0,1}.When *α*_*i*_=1, the computing tasks are offloaded to the MEC server at the edge layer for processing and then processed locally at the terminal device when *α*_*i*_=0; similarly, when *β*_*i*_=1, the computing tasks offloaded to the edge layer will be forwarded to the fog computing node for processing via fiber or microwave transmission when the time comes and still processed at the MEC server when the *β*_*i*_=0. Therefore, the meanings of the main parameters of the developed model are shown in Table 2.

#### 3.1.1. Terminal Layer Model

The arithmetic power of the user terminal device is expressed in terms of the CPU clock cycle frequency *f*_user,*i*_ of the device, and the latency of the task processed at the local user device is as follows:

(1) $$\begin{array}{c} T_{i,\text{exe}}=\frac{C_{i}}{f_{\text{user},i}}. \end{array}$$

In addition, the energy consumption of tasks processed at the local user device is as follows:

(2) $$\begin{array}{c} E_{i,\text{local}}=k_{u}{\left(f_{\text{user},i}\right)}^{2}C_{i}. \end{array}$$

In (2), *k*_*u*_ is the energy factor determined by the built-in CPU chip architecture of the terminal device [23], and by the method of literature [24], the scenario in this study takes *k*_*u*_=10^−27^.

To address the problems of poor terminal standby, high energy consumption, and high latency of video applications in system testing, this study uses energy consumption and latency as the main indicators to reflect the quality of experience (QoE) of users and introduces a latency weighting factor *δ*, which jointly optimizes latency and energy consumption and denotes the total system overhead at the terminal layer as *F*_*i*,local_. In practical application, it can be adjusted according to different service types and optimization requirements, and the delay weighting factor *δ* is increased for delay-sensitive services to obtain better optimization of delay index, and the energy consumption weighting factor is 1 − *δ* and vice versa. Then, there are

(3) $$\begin{array}{c} F_{i,\text{local}}=\delta T_{i,\text{local}}+\left(1-\delta \right)E_{i,\text{local}}. \end{array}$$

When the computing power of the terminal device cannot meet the computing demand, the terminal device transmits the computing task through the uplink to the MEC server at the edge layer for processing, and the existing mainstream large bandwidth wireless communication technologies LTE, 5 G, and WIFI 6 all adopt orthogonal frequency division multiple access (OFDMA) technology, and the data can be transmitted between the uplink channel subcarriers in parallel without interference, so the transmission rate of the user terminal *i* in the sub-band *w*_*i*_ is as follows:

(4) $$\begin{array}{c} R_{i}^{j}=w_{i}\log_{2}\left(1+\frac{P_{i}H_{i}^{j}}{\sigma^{2}}\right). \end{array}$$

In (4), *w*_*i*_ is the sub-band bandwidth of the uplink transmission of the terminal, *P*_*i*_ is the transmit power of the user terminal *i*, *H*_*i*_^*j*^ is the channel gain between the terminal *i* and the edge node *j*, and *σ*^2^ denotes the background noise variance, and then, the offload transmission delay with the device *i* is as follows:

(5) $$\begin{array}{c} T_{i\text{,trans}}^{j}=\frac{D_{i}}{R_{i}^{j}}. \end{array}$$

#### 3.1.2. Edge Layer Model

There are mainly 3 links for computing task offloading to the edge layer: uplink transmission, task processing, and result feedback. After the computing task completes the computing processing, then after coding and compression, the data volume is already very small, and the downlink transmission rate is much higher than the uplink, so the delay of the result feedback link is negligible, and then, the total delay of computing task offloading to the edge layer to complete the task delivery is as follows.

(6) $$\begin{array}{c} T_{i,\text{mec}}^{j}=T_{i\text{,trans}}^{j}+T_{j,\text{exe}}, \end{array}$$

where *T*_*j*,exe_ is the processing latency of the task *i* at the MEC server, and the MEC server allocates the corresponding computational resources *f*_*i*,mec_ for the offloaded tasks *i*, so that we have

(7) $$\begin{array}{c} T_{j,\text{exe}}=\frac{C_{i}}{f_{i.\text{mec}}},\\ s.t.\overset{n}{\underset{i=1}{\sum }}f_{i,\text{mec}}\leq f_{\text{mec,total}}. \end{array}$$

When the task offload shunt occurs from the terminal layer to the edge layer, the energy consumption is mainly oriented towards the terminal layer devices and can be derived from the terminal device transmit power as follows.

(8) $$\begin{array}{c} E_{i,\text{trans}}=P_{i}T_{i\text{,trans}}^{j}=P_{i}\frac{D_{i}}{R_{i}^{j}}. \end{array}$$

Since MEC servers and FC servers are deployed based on communication transmission nodes and secured by utility power, weak wells, or generators, the total energy consumption of tasks offloaded from the terminal layer to the edge layer considers transmission energy consumption, while the offloading from the edge layer to the fog computing layer does not consider the transmission and processing energy consumption of both, so the total overhead of tasks offloaded to MEC servers is as follows:

(9) $$\begin{array}{c} F_{i\text{mec}}^{j}=\delta T_{i,\text{mec}}+\left(1-\delta \right)E_{i,\text{trans}}. \end{array}$$

#### 3.1.3. Fog Computing Layer Model

When a large number of computing tasks are offloaded to the edge layer, which exceeds the load of the existing MEC server, some of the tasks are offloaded to the fog node for processing by completing the uplink transmission through the edge node and the base station relying on the fog node, and the fog node allocates computing resources *f*_*i*,fog_ for processing according to the task load, which includes the transmission delay *T*_*i*,trans_^*j*^ from the terminal to the edge node, the transmission delay *T*_*j*,trans_^*k*^ from the edge node to the fog node, and the processing delay *T*_*k*,exe_ of the fog node, and the total delay of the task offloading to the fog node for processing is as follows:

(10) $$\begin{array}{c} T_{i\text{,fog}}^{k}=T_{i,\text{trans}}^{j}+T_{j,\text{trans}}^{k}+T_{k,\text{exe}}, \end{array}$$

where the transmission between base stations is via optical fiber with a fixed transmission rate *R*_*j*_^*k*^, and then, we have the following:

(11) $$\begin{array}{c} T_{j,\text{trans}}^{k}=\frac{D_{i}}{R_{j}^{k}}. \end{array}$$

The processing delay of the fog node is as follows:

(12) $$\begin{array}{c} T_{k,\text{exe}}=\frac{C_{i}}{f_{i,\text{fog}}},\\ s.t.\sum_{i=1}^{n}f_{i,\text{fog}}\leq f_{\text{fog,total}}. \end{array}$$

Also, in this offload mode, only the transmission energy consumption of the end layer devices is counted, so the total offload overhead is as follows:

(13) $$\begin{array}{c} F_{i\text{,fog}}^{k}=\delta T_{i,\text{fog}}^{k}+\left(1-\delta \right)E_{i,\text{trans}}. \end{array}$$

Based on the foregoing, the total overhead of the proposed system in this study for processing computational tasks is the weighted sum of the overheads of the processing tasks at each layer.

(14) $$\begin{array}{c} F_{\text{system}}=\sum_{i=1}^{n}\left(1-\alpha_{i}\right)F_{i,\text{local}}+\alpha_{i}\left(1-\beta_{i}\right)F_{i,\text{mec}}^{j}+\alpha_{i}\beta_{i}F_{j,\text{fog}}^{k}. \end{array}$$

### 3.2. Questions to Ask

To further optimize the effectiveness of the proposed “cloud, fog, edge, and end” four-layer system architecture, this study sets the goal of reducing latency and energy consumption, based on a heuristic algorithm to jointly optimize the offloading decision, communication, and computing resource allocation problem, and according to the above model, the offloading overhead represents the weighted sum of latency and energy consumption; therefore, from (14), to reduce the total offloading overhead of the target system, we can obtain problem *Q*.

(15) $$\begin{array}{c} \underset{\alpha ,\beta ,f_{\text{mec}},f_{\text{fog}},w_{i}}{\min}F_{\text{system}}, \end{array}$$

(16) $$\begin{array}{c} \alpha_{i},\beta_{i}\in \left\{0,1\right\},\;\forall i\in n, \end{array}$$

(17) $$\begin{array}{c} \sum_{i=1}^{n}f_{i,\text{mec}}\leq f_{\text{mec,total}}, \end{array}$$

(18) $$\begin{array}{c} 0\leq f_{i,\text{mec}}\leq f_{\text{mec,total}}, \end{array}$$

(19) $$\begin{array}{c} \sum_{i=1}^{n}f_{i,\text{fog}}\leq f_{\text{fog,total}}, \end{array}$$

(20) $$\begin{array}{c} 0\leq f_{i,\text{fog}}\leq f_{\text{fog,total}}, \end{array}$$

(21) $$\begin{array}{c} \sum_{i=1}^{n}w_{i}\leq W, \end{array}$$

(22) $$\begin{array}{c} 0\leq w_{i}\leq W. \end{array}$$

In the above model, *α*_*i*_={*α*_1_, *α*_2_, *α*_3_,…, *α*_*n*_} and *β*_*i*_={*β*_1_, *β*_2_, *β*_3_,…, *β*_*n*_}. The constraints in formulas (16)–(20) correspond to the communication resource allocation problem, indicating that the communication channels are allocated for the end devices during offloading and the sub-bands do not exceed the total bandwidth; (17)–(20) correspond to the computational resource allocation problem, indicating that the computational resources are allocated for the tasks offloaded to the edge nodes or fog nodes and do not exceed the maximum server limit; (16) corresponds to the offloading decision, indicating that there are 2^*n*^ kinds of decisions for each layer when offloading is performed at the terminal and edge layers.

In addition, the two offload decision variables are binary arrays, so the offload decision is a nonlinear restricted 0–1 planning problem, while the communication and computational resource allocation problems have a large number of variables with linear relationships between the user tasks, and the literature [25] has shown that the underlying problem for the joint optimization of computational offload, communication, and computational resource allocation is a mixed integer nonlinear programming (MINP) problem, which is difficult to find the optimal solution, so the complex problem is decomposed into a low-complexity problem to solve.

### 3.3. Question Breakdown

The complex problem is decomposed into multiple equivalent problems using the Tammer decomposition [26] to decouple the relevant variables and then solved by a heuristic algorithm, so that (15) is decomposed as follows:

(23) $$\begin{array}{c} \underset{f_{\text{mec}},f_{\text{fog}},w_{i}}{\min}\left(\underset{\alpha ,\beta }{\min}F_{\text{system}}\right),\\ s.t.\left(18\right)-\left(24\right). \end{array}$$

Let *F*_system_′ correspond to the offloading decision problem in parentheses, and we have the following:

(24) $$\begin{array}{c} F_{\text{system}}^{\prime }=\underset{\alpha ,\beta }{\min}F_{\text{system}},\\ s.t.\left(18\right). \end{array}$$

Meanwhile, question *Q* can be formulated as follows.

(25) $$\begin{array}{c} \underset{f_{\text{mec}},f_{\text{fog}},w_{i}}{\min}F_{\text{system}}^{\prime },\\ s.t.\left(19\right)-\left(24\right). \end{array}$$

In this way, problem *Q* is decomposed into two subproblems, i.e., the offloading decision and the resource allocation problem, and the constraints of the two subproblems are decoupled. In addition, by replacing the decision variables, it can be further simplified *F*_system_′ that the offloading decision variables *α*_*i*_=0 of the terminal layer, the offloading decision variables *α*_*i*_=0 of the edge layer and *β*_*i*_=0, and the offloading decision variables *α*_*i*_=1 of the fog computing layer and *β*_*i*_=1. Finally, the problem is transformed into a convex optimization problem and solved by applying the algorithm proposed in this study.

## 4. Algorithm Improvement and Validation Experiments

### 4.1. Algorithm Improvement Ideas

Among many intelligent optimization algorithms, particle swarm optimization (PSO) [27] is relatively well developed, although it still lacks strict mathematical derivation in some parameter values and relies on scenario-specific experiments and existing research experience to set them, PSO does not rely on gradient and curvature, but performs parallel search based on population intelligence, so for the system proposed in this study that contains a large number of equipment tasks, it can find the optimal or suboptimal solution relatively quickly. However, the classical PSO algorithm is prone to local optimality when dealing with practical problems, and it adopts a randomized approach in population initialization, and the potential of particle ergodicity is not deeply explored, so it is difficult to achieve an optimal balance between the pre-exploration and post-exploitation capabilities of the algorithm.

To this end, many scholars in academia have conducted fruitful research on algorithm improvement. Wang and Liu [28] proposed the concept of population evolution dispersion and a nonlinear dynamic adaptive inertia weight PSO algorithm (inertial particle swarm optimization, IPSO) based on sigmoid function; Wu et al. [29] proposed an adaptive particle swarm algorithm (curves increasing particle swarm optimization, CIPSO) with a curve-increasing strategy; and Yan et al. [30] introduced simulated annealing operation and proposed an adaptive simulated annealing particle swarm algorithm (adaptive simulated annealing particle swarm optimization, ASAPSO) based on hyperbolic tangent function. In addition to the above three improved algorithms all change the balance between global exploration and local exploitation of the algorithm by controlling parameters, the literature [30] also introduced simulated annealing operation to enhance the ability of the algorithm to jump out of the local optimum, which effectively improves the performance of the classical particle swarm algorithm.

To further explore the optimization potential of PSO algorithm, this study improves PSO from four aspects and proposes chaotic teach and learn particle swarm optimization (CTLPSO), which firstly improves the population initialization using sine chaos mapping to improve the blindness caused by random. Secondly, the “teach” phase of the teaching algorithm is introduced into the PSO speed update formula, and the “global exploration” and “local development” capabilities of the algorithm are enhanced. At the same time, to enhance the adaptiveness of the algorithm's “global exploration” and “local exploitation” capabilities, the constant inertia weights are replaced by segmented inertia weights to give the algorithm adaptive capabilities, so that the global optimum, the historical optimum, and the population average act together on the particles and have a comprehensive impact on their displacement direction and step size; finally, the “learning” phase of the teaching algorithm is conducted on the particle population to improve the algorithm's efficiency. “Learning” stage of the particle population is conducted to improve the ability of jumping out of the local optimum in the late iteration of the algorithm.

### 4.2. Chaotic Teaching and Learning Particle Swarm Optimization

#### 4.2.1. Particle Swarm Optimization

The optimization-seeking process of PSO is completed by the particle simulating the foraging flight of a flock of birds, and the displacement of the particle in each iteration consists of three parts, which are the inheritance of the previous velocity, its own learning, and the information interaction of the population, and its velocity and position update equations are as follows.

(26) $$\begin{array}{c} v_{i}\left(k+1\right)=\omega v_{i}\left(k\right)+c_{1}r_{1}\left[P_{\text{best},i}\left(k\right)-x_{i}\left(k\right)\right]\\ +c_{2}r_{2}\left[G_{\text{best}}-x_{i}\left(k\right)\right],\\ x_{i}\left(k+1\right)=x_{i}\left(k\right)+v_{i}\left(k+1\right), \end{array}$$

where *ω* is the inertia weight coefficient; *c*_1_, *c*_2_ are the self-cognitive factor and social cognitive factor, which are important parameters to control the PSO iteration, respectively. *x*_*i*_(*k*) and *v*_*i*_(*k*) represent the position and velocity of the particle *i* at the iteration *k*, respectively; *r*_1_ and *r*_2_ are the random coefficients; *P*_best,*i*_ is the individual optimal position of the particle *i*; and *G*_best_ is the population optimal position.

#### 4.2.2. Teaching-Learning-Based Optimization (TLBO)

Teaching-learning-based optimization (TLBO) [31] simulates a class-based learning approach in which the improvement of the level of the students in the class is guided by the teacher's “teaching,” and at the same time, the students need to “learn” from each other to facilitate the absorption of knowledge. Students need to “learn” from each other to facilitate the absorption of knowledge. The teacher and the learners are equivalent to individuals in an evolutionary algorithm, where the teacher is one of the best adapted individuals and each learner learns a subject that is equivalent to a decision variable. This is defined as follows:

1. Search Space: the search space can be expressed as *S*={*X|x*_*i*_^*L*^ ≤ *xi* ≤ *x*_*i*_^*U*^,  *i*=1,2,…, *d*}, *d* indicates the spatial dimension (the number of decision variables), and *x*_*i*_^*L*^ and *x*_*i*_^*U*^(*i*=1,2,…, *d*) are the upper and lower bounds of each dimension, respectively.
2. Search Point: set *X*_*j*_=(*x*_1_^*j*^, *x*_2_^*j*^,…, *x*_*d*_^*j*^), (*j*=1,2,…, *NP*) is a point *j* in the search space, *x*_*i*_^*j*^(*i*=1,2,…, *d*) is one of the decision variables of point *X*_*j*_, and *NP* is the number of spatial search points, i.e., the number of potential solutions.
3. Class: the set of all points in the search space of TLBO is called class.
4. Students: a point in the class *X*^*j*^=*x*_1_^*j*^, *x*_2_^*j*^,…, *x*_*d*_^*j*^ is called as a student.
5. Teacher: the student *X*_best_ with the best performance in the class is called the teacher and is denoted by *X*_teacher_.

*(1) “Teaching” Phase.* In the “teach” phase of the TLBO algorithm, each student in the class is denoted by *X*_*j*_(*j*=1,2,…, *NP*). To make continuous progress and close the gap between themselves and the teachers, learning is based on the variability between *X*_teacher_ and student averages Mean.

**Figure 4:**
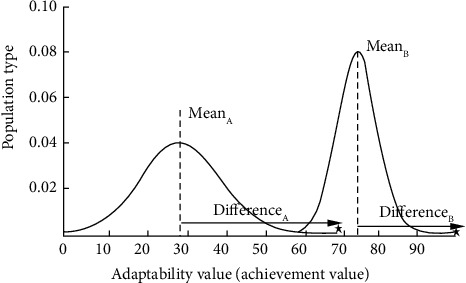
TLBO process model.

As shown in Figure 4, the average class grade in the initial stage is Mean_*A*_=28. The average grade was low and widely distributed, but after several sessions of instruction, the average grade was raised to Mean_*B*_=74, and the distribution of grades was gradually concentrated, indicating that the grades improved through “teaching” and “learning,” and the span of the best and worst grades was reduced. In the “teaching” phase, each student learned from the teacher by taking advantage of the difference in level between the teacher *X*_teacher_ and the average of the student's grades Mean, as follows.

(27) $$\begin{array}{c} X_{\text{new}}^{i}=X_{\text{old}}^{i}+\text{Difference}, \end{array}$$

(28) $$\begin{array}{c} \text{Difference}=r_{i}.\left(X_{\text{teacher}}-TF_{i}.\text{Mean}\right). \end{array}$$

*X*_old_^*i*^ and *X*_new_^*i*^, respectively, indicate the values of the *i* student before and after learning, Mean=1/*NP*∑_*i*=1_^*NP*^*X*^*i*^ is the average of all students, and there are two key parameters: the teaching factor *TF*_*i*_=round[1+rand(0,1)] and the learning step *r*_*i*_=rand(0,1).

*(2) “Learning” Stage.* In the “learning” phase, for each student *X*^*i*^(*i*=1,2,…, *NP*), a learning target *X*^*j*^(*j*=1,2,…, *NP*, *j* ≠ *i*) is randomly selected in the class. *X*^*i*^ makes learning adjustments by analyzing the differences between himself and *X*^*j*^. The learning improvement method is similar to the differential evolution algorithm, except that the learning step *r* in TLBO uses a different learning factor for each student. The “learning” process is implemented using (27).

(29) $$\begin{array}{c} X_{\text{new}}^{i}=\left\{\begin{array}{c} X_{\text{old}}^{i}+r_{i}.\left(X^{i}-X^{j}\right),f\left(X^{j}\right)<f\left(X^{i}\right),\\ X_{\text{old}}^{i}+r_{i}.\left(X^{j}-X^{i}\right),f\left(X^{i}\right)<f\left(X^{j}\right). \end{array}\right\} \end{array}$$

In (29), the *r*_*i*_=*U*(0,1) denotes the learning factor of the student *i*, i.e., the learning step.

*(3) Update Method*. The update operation is performed separately when the learner goes through the “teaching” and “learning” phases. The updating idea is similar to the differential evolution algorithm. If individual *X*_new_^*i*^ after learning is better than student *X*_old_^*i*^ before learning, *X*_old_^*i*^ is replaced with *X*_new_^*i*^ . Otherwise, *X*_old_^*i*^ is kept unchanged and updated in the following way.

 
If*X*_new_^*i*^isbetterthan*X*_old_^*i*^
 
*X*_old_^*i*^=*X*_new_^*i*^
 
Endif

#### 4.2.3. Algorithm Improvement Innovation

*(1) PSO's Sine Chaos Initialization*. The PSO population initialization uses a pseudorandom sequence, although the population of traversalism is guaranteed, but the performance of the chaotic sequence is initialized, crossover and variation use of the chaotic sequence is often better. Sine mapping is a typical representative of chaotic mapping, and its mathematical form is as follows:

(30) $$\begin{array}{c} x_{k+1}=\frac{4}{a}\sin\left(\pi x_{k}\right),a\in \left(0,4\right]. \end{array}$$

**Figure 5:**
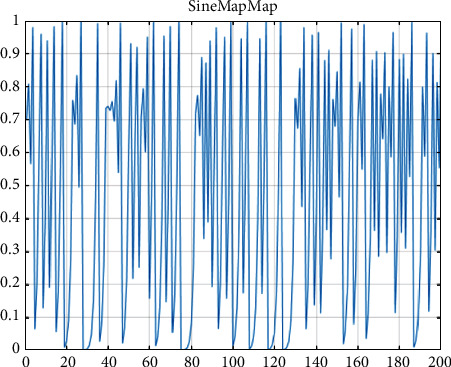
Distribution of sine mapping iterations.

The range of *x* in the sine expression is [0,1]. The distribution of 200 iterations of the sine mapping is shown in Figure 5.

As can be seen from Figure 5, the sine mapping is distributed between [0,1], and the chaotic property is used instead of random initialization, which can make the population more uniformly distributed in the search space and improve the efficiency of population exploration.

*(2) Segmented Inertia Weights*. In classical PSO, the inertia weights are constant and the algorithm inherits a constant speed for its own history, which does not allow the flexibility to adapt the algorithm performance to different problems and different search phases. For this reason, an adaptive mechanism is introduced so that the inertia weights *ω* are varied by segments, with the following expression.

(31) $$\begin{array}{c} \omega =\left\{\begin{array}{cc} \frac{1-t}{0.7\cdot \text{MaxIter}}, & \text{if}t\leq 0.7\cdot \text{MaxIter,}\\ \frac{10}{3}-\frac{t}{0.3\cdot \text{MaxIter}}, & \text{else} \end{array}\right\}, \end{array}$$

*(3) Speed Update into the “Teaching” Phase.* By introducing the TLBO “teach” phase into the PSO, the speed update equation is improved to

(32) $$\begin{array}{c} v_{i}\left(k+1\right)=\omega v_{i}\left(k\right)+c_{1}r_{1}\left(P_{\text{best},i}\left(k\right)x\left(k\right)\right)\\ +c_{2}r_{2}\left(G_{\text{best}}-x_{i}\left(k\right)\right)\\ +c_{3}r_{3}\left(r_{i.}\left(G_{\text{best}}-TF_{i.}X\text{Mean}\right)\right), \end{array}$$

where *c*_1_=*c*_2_=*c*_3_=1/3, and *X*_Mean_ is the mean value of the population.

*(4) Population Renewal Based on the “Learning” Phase*. Learning steps in TLBO: the “learning” process is achieved by applying different learning factors to each student.

(33) $$\begin{array}{c} X_{\text{new}}^{i}=\left\{\begin{array}{cc} X_{\text{old}}^{i}+r_{i.}\left(X^{i}-X^{j}\right), & f\left(X^{j}\right)<f\left(X^{i}\right),\\ X_{\text{old}}^{i}+r_{i.}\left(X^{j}-X^{i}\right), & f\left(X^{i}\right)<f\left(X^{j}\right). \end{array}\right\} \end{array}$$

**Figure 6:**
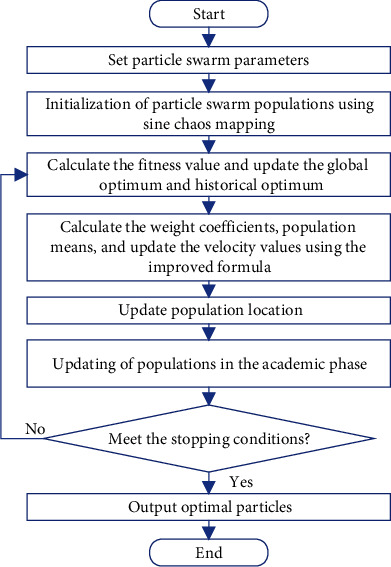
Algorithm flow chart.

In equation (33), *r*_*i*_=*U*(0,1) is the learning step of student *i*. The CTLPSO algorithm flow is shown in Figure 6.

### 4.3. Algorithm Performance Comparison Experiment

In this section, performance verification and comparative analysis of the TLIPSO algorithm are performed based on the simulation environment of HUAWEI MateBook 14 with Intel Core i5 1.6 GHz processor and 16 GB of memory, operating system of Windows 10 Professional 64 bit, and using MATLAB 2018b as the simulation platform.

**Figure 7:**
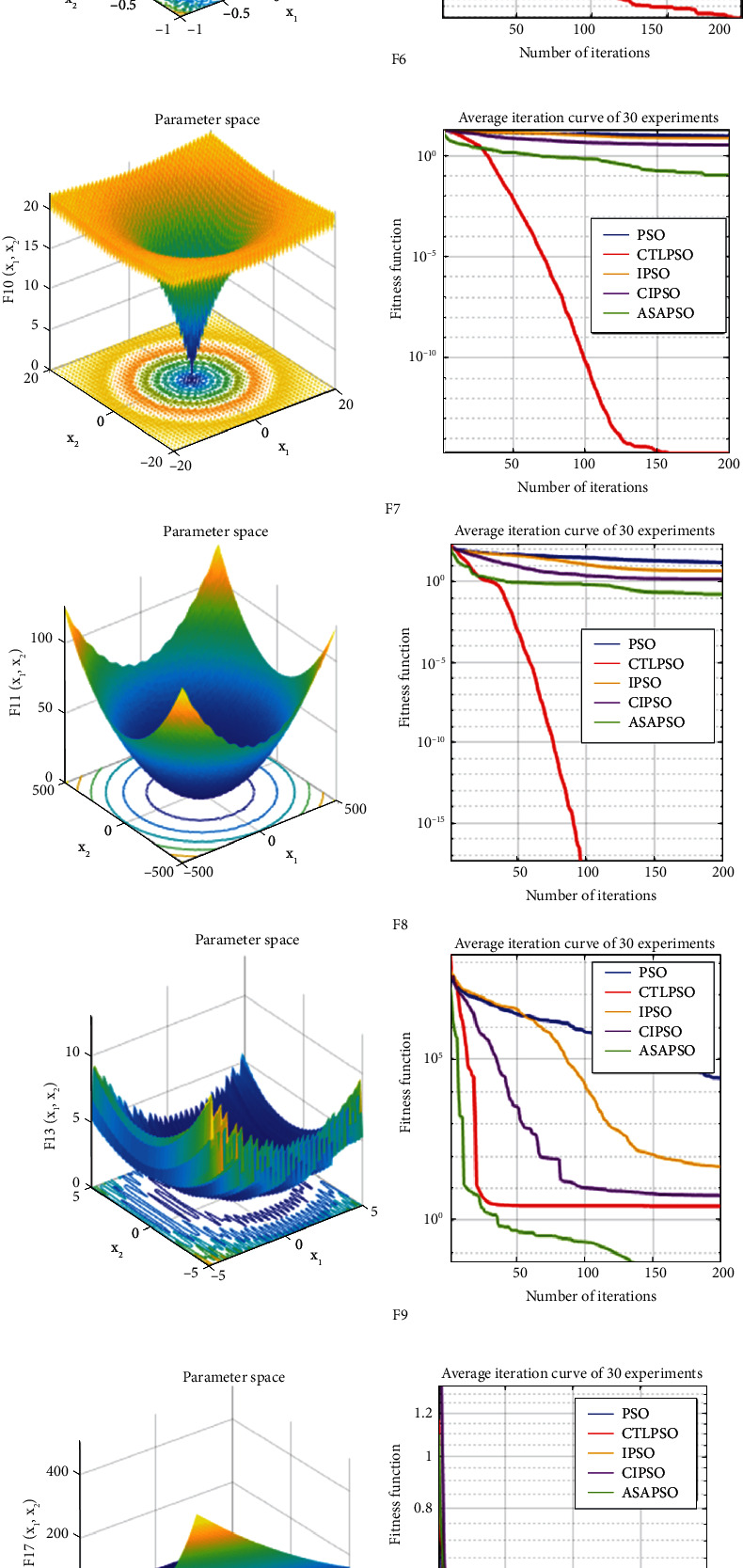
Graph of experimental results.

To verify the performance of the algorithm and ensure the generality and test integrity of the algorithm, based on the 11 test benchmark functions shown in Table 3, the CTLPSO proposed in this study is carried out with classical PSO, IPSO [28], CIPSO [29], and ASAPSO [30] for simulation comparison experiments, setting the number of populations to 30, the maximum number of iterations to 200, and running 30 times continuously, respectively. The optimal fitness value (best), the average fitness value (mean), the worst fitness value (worst), the standard deviation (std), and the total running time (time) of each algorithm are recorded in Table 4. In addition, the comparison of the test functions and algorithm curves is shown in Figure 7, where *F*10 (a) and *F*10 (b) are local enlargements of *F*10, and *F*11 (a) and *F*11 (b) are local enlargements o*f F*11.

#### 4.3.1. Comparison of Algorithm Convergence Accuracy

From the optimization search results, the CTLPSO outperforms other algorithms in all the tested functions except for the suboptimal solutions in functions F5 and F9. In F1-F4 and F6-F8, the optimal solution is obtained with a great advantage, and the optimization improvement is up to 50 times compared with other algorithms. In F10 and F11, the highest convergence accuracy is achieved with a slight advantage.

#### 4.3.2. Algorithm Convergence Speed Comparison

Figure 7 shows the optimization curve plots for the 11 tested functions, and the experimental results are broadly classified into 3 categories based on the average curve changes of 200 iterations per round for 30 consecutive rounds of experiments.

- The convergence speed of the algorithm is in the 2nd place in the early iterations (the first 25 iterations on average) and then reverses to become the 1st, and the convergence accuracy leads by a great margin. (b) The conditions are met for *F*1, *F*2, *F*4, and *F*6-8.
- The convergence rate is always in the 1st position. The condition is met by *F*3.
- The convergence speed is in the 2nd place with a small disadvantage in the early stage, and then, the convergence accuracy is in the 2nd place and falls into the local optimum, which contains *F*5 and *F*9.
- The convergence speed is basically equal to other algorithms at the beginning of the iteration (average first 6 iterations), and then, it overtakes in the 1st with a slight advantage, which is met by *F*10 and *F*11. In summary, the CTLPSO algorithm proposed in this study has a strong advantage in the convergence speed.

#### 4.3.3. Algorithm Stability Comparison

The standard deviation of the experimental results shows that CTLPSO results have the smallest standard deviation among a total of 9 test functions F1-F8 and F10 and have an exponential advantage of up to 1054 times, while the stability ranks 2nd and 3rd in functions F9 and F11, respectively. Overall, CTLPSO has strong search stability.

#### 4.3.4. Algorithm Complexity Comparison

To enhance the ability of PSO algorithm to deal with high-dimensional complex problems and jump out of local optimum, CTLPSO and ASAPSO integrate the update mechanism of TLBO and SA algorithms, respectively, in addition to improving the algorithm parameters and introducing the adaptive mechanism. From the level of algorithm design architecture, compared with other algorithms, the depth-improved algorithm has increased complexity compared with the classical PSO algorithm and the light-weight-improved algorithm, but the CTLPSO algorithm proposed in this study achieves a better balance between dealing with complex high-dimensional problems and reducing the complexity of the algorithm. Therefore, the CTLPSO algorithm in this study achieves a lower algorithm complexity on the basis of the guaranteed algorithm-seeking performance complexity.

### 4.4. Conclusion

In summary, in most cases, the CTLPSO algorithm proposed in this study has an obvious advantage in convergence accuracy, with a maximum difference of 50 times, and in convergence speed, with a slight disadvantage in the 2nd place at the beginning of the iteration, and then achieves a speed reversal, and has an obvious advantage in algorithm stability, with a maximum advantage of times. There are many innovative points, both carry out the improvement of parameters, the introduction of adaptive mechanism and the fusion operation of other algorithm update mechanism, and carry out the deep improvement to enhance the algorithm to deal with complex high-dimensional problems and jump out of the local optimum ability, which objectively also increases the algorithm operation process; therefore, from the total call time of each algorithm, the order from simple to complex is PSO, IPSO, CIPSO, and CTLPSO. However, it can be seen from the data that the CTLPSO proposed in this study can control the total time slightly higher than the first three algorithms and much lower than ASAPSO with one more innovation point than ASAPSO, so the algorithm proposed in this study achieves a good balance between improving the algorithm performance and controlling the algorithm complexity.

Overall, the CTLPSO algorithm improves the efficiency of the global exploration of the algorithm's pre-optimization search, improves the disadvantages of PSO's easy premature maturity, and satisfies the needs of the algorithm to deal with high-dimensional complex problems and different stages of the optimization search through the innovations of the population initialization chaos operation, parameter improvement, adaptive mechanism introduction, and update methods, while effectively controlling the complexity of the algorithm.

## 5. Experimental Simulation and Result Analysis

### 5.1. Environment Construction and Parameter Setting

This chapter, also based on MATLAB platform, conducts simulation experiments on the problem presented in Section 3 to compare and analyze the performance of the system architecture, algorithm model, and offloading strategy proposed in this study by controlling the variables, and the simulation parameters are set as shown in Table 5.

### 5.2. Simulation Analysis

#### 5.2.1. System Architecture Performance Verification

**Figure 8:**
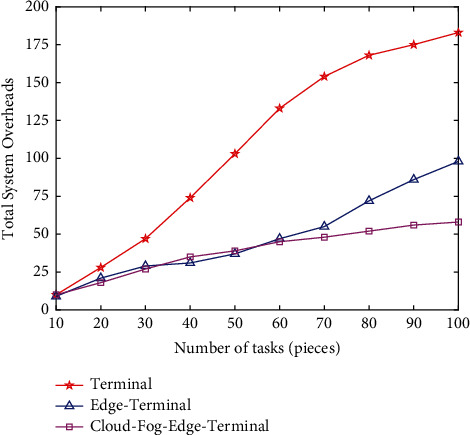
Performance of the number of tasks in different architectures.

Firstly, the proposed “cloud, fog, edge, and end” collaborative architecture is verified by setting different number of tasks to run under three architectures: terminal, edge, and cloud-fog-edge based on random offloading strategy. As shown in Figure 8, when the number of tasks is small, the total overhead of processing tasks of the three architectures is not much different, and as the number of tasks increases, the terminal side cannot meet the processing demand of a large number of tasks, and the growth of delay and energy consumption makes the total system overhead increase nearly linearly and more. The cloud-side architecture has no obvious advantage in a small number of tasks, and the total overhead is slightly higher than the edge-side architecture in a medium task volume of 34–53, because the computational tasks are based on random offloading strategy, and the increase in transmission path makes the channel quality and other uncertainties increase accordingly, and when the channel quality is unstable, there is a possibility of offloading to the fog node or even the central cloud, which causes additional delay and energy consumption. In contrast, the system has obvious advantages when facing large task volumes.

#### 5.2.2. Offloading Performance Analysis

**Figure 9:**
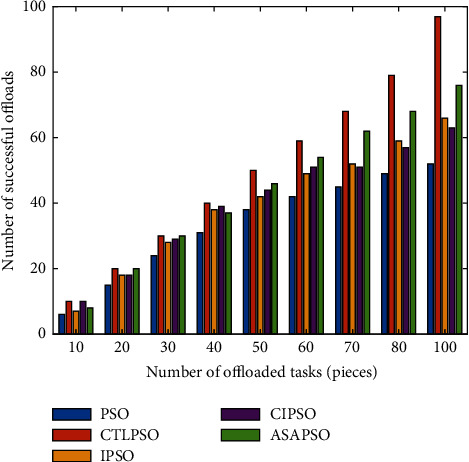
Effect of the number of tasks on the success rate of uninstallation.

Because of the limited resources at the edge layer, with the surge in the number of users and data traffic, when the available resources at the edge layer are insufficient, then it needs to be forwarded to the fog computing layer for processing. Different offloading strategies have different impacts on the addressing and resource allocation of computing tasks, which directly affect the success rate of offloading, so the actual PSO, CTLPSO, IPSO, CIPSO, and ASAPSO-based algorithms are compared and analyzed for number of offloading tasks. As shown in Figure 9, when the task volume is small, the offloading success rates of the five algorithm strategies do not differ much because the available resources are abundant, while when the task volume continues to grow, the competition for communication and computing resources among offloading users also continues to rise, and the complexity of the offloading strategy and resource allocation mechanism will also affect the offloading performance when the user offloading behavior is extended from 2 layers at the edge end to 4 layers at the cloud-edge end, and from the simulation results, when the task exceeds 50, the algorithm proposed in this study has obvious advantages.

#### 5.2.3. Impact of the Number of Iterations on the System Overhead

**Figure 10:**
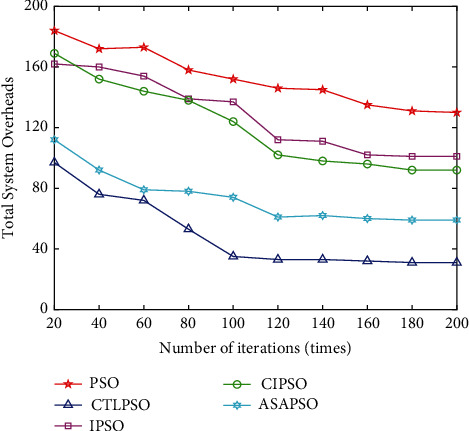
Effect of the number of iterations of different algorithms on the system overhead.

Figure 10 compares the convergence performance of PSO, CTLPSO, IPSO, CIPSO, and ASAPSO. In the early iterations, the convergence trend of the remaining four algorithms is relatively flat, while the convergence trend of the proposed CTLPSO is faster, and it is close to the optimal solution at about the 100th iteration. The global exploration efficiency in the early stage of the algorithm is higher. In addition, all the five algorithms have a short period of no fluctuation in the early curve and then return to normal convergence, which indicates that all of them have the ability to jump out of the local optimum. Overall, CTLPSO has significant advantages over PSO, IPSO, and CIPSO in terms of convergence speed and accuracy, and there is not much difference in convergence speed compared with ASAPSO, and CTLPSO converges to the optimal solution at 100 iterations and has obvious advantages in convergence accuracy.

#### 5.2.4. Impact of the Number of Tasks on System Overhead

**Figure 11:**
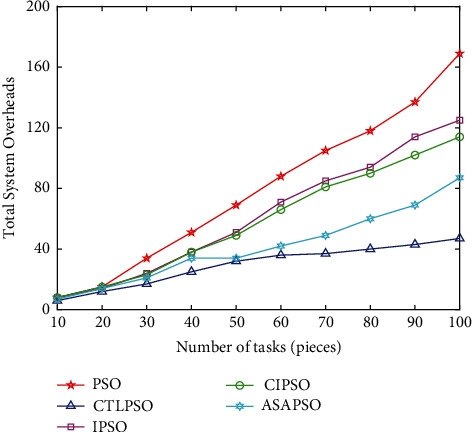
Impact of number of tasks on system overhead.

Figure 11 compares the changes in the total system overhead of the 5 algorithms with increasing task volume. When the task volume is small, the 5 algorithms do not differ much because they can meet the task processing demand at the terminal side, and when the task volume increases, the task processing scope is at the edge range, so the CTLPSO advantage is not obvious and is close to ASAPSO, but as the task volume continues to increase and the scope expands to the fog layer, the complexity of the ASAPSO algorithm will generate additional delay and thus increase the system overhead, while the CTLPSO proposed in this study maintains a good balance of convergence and algorithm complexity in the face of complex multidimensional problems, and the advantage gradually expands when the number of tasks is greater than 60.

## 6. Conclusion

The large number of computation-intensive and latency-sensitive applications poses many challenges to information system architecture, bearer network, and cloud computing technology. Although MEC effectively complements and enhances the information service capability of cloud computing at the edge and achieves the goals of “reduced latency, bandwidth saving, high isolation, load sharing, and strong awareness,” it still faces the problems of limited resources and high deployment costs. To this end, this study proposes a four-layer architecture of “cloud, fog, edge, and end,” a joint optimization model for offload decision and resource allocation, and a chaotic teaching particle swarm algorithm. Through experimental verification, the proposed four-layer architecture can effectively improve the offloading success rate and reduce the total system overhead of computational offloading. This study can be used as an idea to solve the resource-constrained problem of MEC. In the future, with the improvement of the industry chain and the reduction in the cost of MEC devices, the problem of resource saturation can likewise be solved by means of lightweight MEC clustering deployment.

## Figures and Tables

**Table 1:** Computational task processing model.

Unload location	Trigger conditions
Local devices	Low task volume, local devices can do the task themselves or there are not enough resources available on the edge layer servers
Edge layer nodes	MEC server resources are fully available and the terminal is unable to complete task processing efficiently
Fog layer node	Task volume is too large for local devices to execute alone, and the edge nodes do not have sufficient available resources, so the tasks are offloaded to MEC and then forwarded to fog nodes for collaborative processing
Data center	The task volume is very large and the latency requirement is not high, and there are not enough computing resources in the edge layer and fog layer, or the content-based services do not have the corresponding resources for distribution and storage in the edge layer and fog layer

**Table 2:** Main parameter symbol meaning.

Parameters	Meaning
*D*_*i*_	Input data volume
*C*_*i*_	The CPU cycles required to complete the first task
*T*_*i*,local_	Task processing latency at the local end device
*f*_user,*i*_	CPU clock cycle frequency of terminal device
*E*_*i*,local_	Calculated energy consumption for local devices
*k*_*u*_	End device energy factor
*δ*	Time delay optimization weighting factor
*F*_*i*,local_	Total task local processing overhead
*W*	Uplink bandwidth
*w*_*i*_	Task *i* uplink sub-band bandwidth
*P*_*i*_	The terminal *i* device transmits power
*H*_*i*_^*j*^	Channel gain between terminal device *i* and edge node *j*
*σ* ^2^	Background noise variance
*R*_*i*_^*j*^	Transmission speed of terminal *i* to edge node *j*
*T*_*i*_^*j*^ _,trans_	Upstream transmission delay of the device *i*
*T*_*j*,exe_	Processing delay on the MEC side
*T*_*i*_^*j*^ _,mec_	Task *i* total latency of unload to MEC processing
*f*_*i*,*mec*_	MEC server's allocated computing power resources for tasks *i*
*f*_mec,total_	MEC total computing resources
*E*_*i*,trans_	Task *i* offload transmission energy to MEC
*F*_*i*_^*j*^ _,mec_	Task *i* offload to edge layer processing total overhead
*T*_*j*_^*k*^ _,trans_	Task *i* uplink transmission delay from edge node to fog node
*T*_*k*,exe_	Processing latency of fog computing nodes
*T*_*i*_^*k*^ _,fog_	Task *i* total delay for offload to fog node processing
*R*_*j*_^*k*^	Uplink transmission delay from edge node to fog node
*f*_*i*,fog_	Fog computing nodes allocate computing power resources for tasks *i*

**Table 3:** Set of test functions.

Number	Function	Dim	Range	*f*_min_
*F*1	*F*_1_(*x*)=∑_*i*=1_^*n*^*x*_*i*_^2^	30	[−100, 100]	0
*F*2	*F*_2_(*x*)=∑_*i*=1_|*x*_*i*_|+∏_*i*=1_^*n*^|*x*_*i*_|	30	[−10, 10]	0
*F*3	*F*_3_(*x*)=∑_*i*=1_(∑_*j*−1_^*i*^*x*_*j*_)^2^	30	[−100, 100]	0
*F*4	$F_{4}\left(x\right)=\underset{i}{\max}\left\{\left|x_{i}\right|,1\leq i\leq n\right\}$	30	[−100, 100]	0
*F*5	*F*_5_(*x*)=∑_*i*=1_^*n*−1^[100(*x*_*i*+1_ − *x*_*i*_^2^)^2^+(*x*_*i*_ − 1)^2^]	30	[−30, 30]	0
*F*6	*F*_6_(*x*)=∑_*i*=1_^*n*^*ix*_*i*_^4^+random[0,1)	30	[−1.28, 1.28]	0
*F*7	$F_{7}\left(x\right)=-20\;\exp\left(-0.2\sqrt{1/n\sum_{i=1}^{n}x_{i}^{2}}\right)-\exp\left(1/n\sum_{i=1}^{n}\cos\left(2\pi x_{i}\right)\right)+20+e$	30	[−32, 32]	0
*F*8	$F_{8}\left(x\right)=1/4000\sum_{i=1}^{n}x_{i}^{2}-\prod_{i=1}^{n}\cos\left(x_{i}/\sqrt{i}\right)+1$	30	[−600, 600]	0
*F*9	$\begin{array}{c} F_{9}\left(x\right)=0.1\left\{\sin^{2}\left(3\pi x_{i}\right)+\sum_{i=1}^{n}{\left(x_{i}-1\right)}^{2}\left[1+\;\sin^{2}\left(3\pi x_{i}+1\right)\right]+{\left(x_{n}-1\right)}^{2}\left[1+\;\sin^{2}\left(2\pi x_{n}\right)\right]\right\}\\ +\sum_{i=1}^{n}u\left(x_{i},5,100,4\right) \end{array}$	30	[−50, 50]	0
*F*10	*F*_10_(*x*)=(*x*_2_ − 5.1/4*π*^2^*x*_1_^2^+5/*πx*_1_ − 6)^2^+10(1 − 1/8*π*)cos *x*_1_+10	2	[−5, 5]	0.398
*F*11	$\begin{array}{c} F_{11}\left(x\right)=\left[1+{\left(x_{1}+x_{2}+1\right)}^{2}\left(19-14x_{1}+3x_{1}^{2}-14x_{2}+6x_{1}x_{2}+3x_{2}^{2}\right)\right]\\ \times \left[30+{\left(2x_{1}-3x_{2}\right)}^{2}\times \left(18-32x_{1}+12x_{1}^{2}+48x_{2}-36x_{1}x_{2}+27x_{2}^{2}\right)\right] \end{array}$	2	[−2, 2]	3

**Table 4:** Population size of 30 maximum 200 iterations per iteration for 30 consecutive experiments.

Function	Algorithm	Optimal fitness value (best)	The optimal solution corresponds to the average fitness value (mean)	Worst adaptation value (worst)	Standard deviation (std)	Total time/sec (time/s)
*F1*	PSO	688.7089	1457.107	2724.1243	504.927	1.427
	CTLPSO	1.0215*e*-55	1.0012*e*-51	1.9587*e*-50	3.5739*e*-51	2.310
	IPSO	115.9884	510.5799	1075.1169	274.0713	1.584
	CIPSO	6.2062	55.0759	159.8691	44.6851	1.691
	ASAPSO	1.9062*e*-05	0.20295	1.9261	0.40706	8.118

*F2*	PSO	11.1739	32.199	94.2118	17.7586	1.494
	CTLPSO	2.0644*e*-28	4.4092*e*-26	2.5927*e*-25	6.7801*e*-26	3.473
	IPSO	8.2146	16.1931	31.5735	6.3284	1.615
	CIPSO	1.0474	4.5607	8.257	1.7571	1.808
	ASAPSO	0.004721	0.17654	0.60898	0.16134	8.348

*F3*	PSO	5954.4538	14428.6526	41658.4972	8213.2678	3.633
	CTLPSO	2.5765*e*-56	2.3859*e*-50	6.5794*e*-49	1.1985*e*-49	7.821
	IPSO	568.288	5400.7559	23845.1894	5275.5004	3.803
	CIPSO	50.7281	1189.4411	7562.3882	1608.3223	3.870
	ASAPSO	0.025663	37.3842	324.5528	79.0064	23.261

*F4*	PSO	9.3672	18.0263	27.5438	4.3495	1.531
	CTLPSO	1.9901*e*-28	6.8895*e*-26	1.5883*e*-24	2.8894*e*-25	3.618
	IPSO	6.7326	12.1719	21.4339	3.762	1.789
	CIPSO	0.95111	3.088	6.5994	1.51	1.847
	ASAPSO	1.3805*e*-06	0.067248	0.3404	0.08106	8.841

*F5*	PSO	40236.5912	249639.6662	829457.2647	201156.6828	1.918
	CTLPSO	28.844	28.9427	28.9771	0.029461	4.423
	IPSO	905.6092	17964.6325	121043.1986	22415.7999	2.056
	CIPSO	132.2292	989.3633	7020.862	1456.4046	2.153
	ASAPSO	0.0027619	1.2898	29.7361	5.3891	11.084

*F6*	PSO	0.19853	2.9565	25.2302	5.1565	2.565
	CTLPSO	1.6319*e*-05	0.0001902	0.00066071	0.00016004	5.576
	IPSO	0.062995	3.5061	21.6622	5.3885	2.770
	CIPSO	0.0038062	0.045352	0.16613	0.037	2.873
	ASAPSO	2.173*e*-06	0.001172	0.0038589	0.0011222	12.687

*F7*	PSO	6.5531	9.3753	11.8192	1.389	1.961
	CTLPSO	8.8818*e*-16	2.0724*e*-15	4.4409*e*-15	1.7034*e*-15	4.162
	IPSO	4.6432	7.4084	19.9668	2.8663	2.064
	CIPSO	1.456	3.0836	4.6343	0.79603	2.161
	ASAPSO	0.00024989	0.10962	1.2343	0.238	10.488

*F8*	PSO	2.8158	16.2781	35.543	7.216	2.102
	CTLPSO	0	0	0	0	4.402
	IPSO	1.7522	4.2216	9.4596	1.6479	2.222
	CIPSO	1.1187	1.7237	3.9132	0.5486	2.390
	ASAPSO	0.00011151	0.15313	1.032	0.24847	11.607

*F9*	PSO	200.2253	82535.0018	652159.7124	146589.7186	4.361
	CTLPSO	2.0832	2.8053	3.5637	0.36512	9.035
	IPSO	10.7825	93.5021	1615.3978	288.6328	4.557
	CIPSO	3.3443	5.9403	11.5836	1.9598	4.539
	ASAPSO	1.6566*e*-12	0.0077261	0.077976	0.016162	22.418

*F10*	PSO	0.39789	0.39791	0.39807	4.1782*e*-05	0.922
	CTLPSO	0.39789	0.39789	0.39791	5.0606*e*-06	2.618
	IPSO	0.39789	0.39789	0.39789	0	1.144
	CIPSO	0.39789	0.39791	0.39801	3.0198*e*-05	1.064
	ASAPSO	0.39789	0.39789	0.39789	0	7.928

*F11*	PSO	3.0001	3.0018	3.0124	0.0025442	0.915
	CTLPSO	3	3	3	4.8911*e*-06	2.770
	IPSO	3	3	3	6.7162*e*-14	1.046
	CIPSO	3	3.0009	3.0039	0.0012	1.053
	ASAPSO	3	3	3	3.5651*e*-15	7.553

**Table 5:** Main parameter settings.

Parameters	Values
*D*_*i*_	100–500 kB take random values
*f*_*i*_	500–1500 cycle/bit
*f*_user,*i*_	0.5–1 GHz take random values
*f*_*i*,mec_	1–100 GHz take random values
*f*_*i*,*fog*_	1–500 GHz take random values
*K*_max_	200
*R*_*j*_^*k*^	0.1 GB/s
*W*	50 MHz
*H*_*i*_^*j*^	2 × 10^−10^−2 × 10^–6^
*σ* ^2^	10^−9^ W
*k*_*u*_	10^–27^
*P*_*i*_	0.1 W
*N*	200
*c*_1_/*c*_2_/*c*_3_	1/3

## Data Availability

The data used to support the findings of this study are available from the corresponding author upon request.
